# Computational fluid dynamics simulation of a jet crystallizer for continuous crystallization of lovastatin

**DOI:** 10.1038/s41598-023-51088-y

**Published:** 2024-01-09

**Authors:** Mohammad Zarei, Hamid Reza Norouzi, Ali M. Sahlodin

**Affiliations:** Department of Chemical Engineering, Amirkabir University of Technology (Tehran Polytechnic), No. 350, Hafez, Tehran, 15875-4413 Iran

**Keywords:** Chemical engineering, Computational science

## Abstract

Continuous crystallization of lovastatin from a lovastatin-methanol solution and water as the anti-solvent in an impinging jet crystallizer is investigated using a computational fluid dynamics model. To capture the important phenomena, the model is coupled with micro-mixing, population balance, and related energy balance equations. It is implemented in OpenFOAM and validated against experimental data, where a fairly good agreement is found. The effects of key process parameters on the crystallization performance are also studied using the validated model. The results show that increasing the inlet jet velocity from 1 to 4 m/s yields a much narrower size distribution and 70% reduction in the mean crystal size. The four-fold increase in the inlet jet velocity also reduces the crystal production rate by one order of magnitude. Also, it is found that increasing the inlet supersaturation ratio from 6.8 to 8.8 nearly doubles the mean crystal size. Moreover, it results in a wider size distribution and a six-fold increase in the crystal production rate. The simulations also confirm that lower solution to anti-solvent mass flow ratios yield a wider size distribution, a larger mean crystal size and a higher crystal production rate. Increasing this ratio from 0.5 to 2 reduces the production rate by two orders of magnitude.

## Introduction

Crystallization is an important separation technique that is used widely in the chemical, food, and pharmaceutical industries. In the pharmaceutical manufacturing, crystallization is performed in a batch manner traditionally. Batch-wise crystallization enables offline testing of the final product. If the product does not meet certain quality criteria such as purity and size distribution, it can subsequently be isolated as off-specification, preventing the propagation of the adverse impact to downstream units. In this sense, batch-wise operation is the preferred choice in the tightly regulated pharmaceutical industry^1^. On the other hand, it is argued that continuous manufacturing can offer higher product consistency, higher safety, lower environmental impact, better scalability^2^, and higher profit^3^. These advantages together with the increasing competition and need for fulfilling the global demand have recently made continuous pharmaceutical manufacturing an attractive choice for both industry and regulatory agencies^4^. To this end, a lot of efforts have been put on transforming batch-wise operation technologies to their continuous counterparts. These efforts have resulted in numerous scholarly publications on different aspects of continuous pharmaceutical manufacturing, early prototypes, and even commercialized continuous manufacturing plants^5,6^.

As a key part of continuous pharmaceutical manufacturing, continuous crystallization has received a lot of attention recently. Mixed-suspension, mixed-product removal (MSMPR) and plug-flow crystallizers (PFCs) are well-studied types of continuous crystallizers^2,7^. Depending on the application, PFCs may be preferred over MSMPRs, as the latter could suffer from the production of larger surface crystals and the possibility of breaking the crystals with the action of the stirrer^7,8^. Other continuous crystallization designs include continuous oscillatory baffled crystallizers (COBCs) and jet crystallizers^9^. The vibration of baffles in the COBC generates cycles of vortices, helping with radial motions and yielding more uniform mixing compared with a conventional PFC^9^. Similar to PFCs, COBCs can suffer from formation of sediment on the walls, making it difficult to clean^8^. On the other hand, impingement jet crystallization is recognized as one of the most reliable methods of producing small crystals with a limited size distribution. The basic principle in this design is the use of intense fluid flow collisions to achieve homogeneous mixing^10,11^.

In terms of the crystallization method, anti-solvent crystallization is a common technique in the pharmaceutical industry^12^. An advantage of anti-solvent crystallization is its ability to crystallize temperature-sensitive drugs without temperature upsets^13^. The narrow particle size distribution is particularly important for inhalable drugs, as a specific size range should be targeted to the human respiratory tract^14,15^. Alvarez and Myerson^2^ used water as anti-solvent to produce flufenamic acid crystals dissolved in ethanol (solvent) and used acetone as the anti-solvent to produce glutamic acid crystals in water in a tubular crystallizer. They observed that by increasing the number of anti-solvent inlets along the crystallizer, crystals with a wider size distribution were produced. Mahajan and Kirwan^11^ measured the dependence of the crystal size distribution (CSD) on the jet velocity and the supersaturation level in the jet crystallizer through anti-solvent crystallization to produce lovastatin, and it was observed that high velocities and low saturation produced smaller crystals. Johnson and Prud’homme^16^ also investigated the dependence of the dimethoxypropane acid crystal size distribution on the Reynolds number of the inlet stream in a jet crystallizer. It was also observed that short residence times caused by high velocities led to crystals of smaller sizes.

A number of simulation studies on continuous crystallization have been published so far. The simulation models typically consist of computational fluid dynamics (CFD) coupled with population balance equations (PBEs) in order to properly capture crystal size distribution (see e.g.^17,18^ for recent examples of CFD-PBE crystallization modeling). Here, those studies focusing on anti-solvent crystallization, preferably of lovastatin and in jet crystallizers, are considered. Woo et al.^19^ developed a CFD-PBE model with micromixing for anti-solvent crystallization in a semi-batch crystallizer and investigated the effects of design parameters on the crystal size distribution (CSD) and growth and dissolution rates. Woo et al.^14^ simulated the CSD in anti-solvent crystallization of lovastatin in a jet crystallizer at different Reynolds numbers. It was reported that the crystal size distribution was wider at lower Reynolds numbers due to longer residence time for nucleation and growth. Pirkle et al.^20^ simulated anti-solvent crystallization of lovastatin in a continuous tubular crystallizer with coaxial mixing. They observed that with changes in the inlet flow nozzle diameter and by keeping the inlet flow rate constant, no significant changes occurred in the particle size distribution. However, by keeping the inlet flow nozzle diameter constant and increasing the inlet flow rate, the particle size distribution became narrower. da Rosa et al.^21^ simulated continuous anti-solvent crystallization of lovastatin in a tubular crystallizer with radial inlets added for enhanced mixing. Their simulations showed that better mixing was achieved with two opposing radial inlets. They also examined the effect of inlet velocity on the particle size distribution and concluded that the particle size distribution became narrower at higher inlet velocities. The idea of a multi-orifice jet crystallizer was also considered in^22^, where the crystallization performance for NaCl was investigated both experimentally and numerically using a CFD-PBE model. Cheng et al.^23^ simulated the anti-solvent crystallization of lovastatin in a jet crystallizer with focus on the numerical solution schemes and crystal size classification type (uniform and geometric) and the physics of a single-phase flow or a mixture model. They showed that, at high jet velocities (i.e., greater than 6 m/s) geometric size classification outperforms the uniform one. Also, single-phase and mixture models result in similar CSDs, especially when the mean crystal size is below 20 μm.

From the above-mentioned CFD-PBE simulation studies, only the references^14,23^ focus on anti-solvent crystallization of lovastatin specifically in a jet crystallizer (without a tubular element). In ref.^14^, the effect of only the Reynolds number on the crystallization performance is studied. In ref.^23^, the focus is more on proposing different modeling approaches (e.g., single- or multi-phase mixture models) and investigating how accurate they are at different jet velocities. The objective in the present work, however, is to perform a rather comprehensive study of the impacts of key operating parameters on the main process performance criteria including the nucleation, growth, CSD, and production rate. The operating parameters considered are inlet supersaturation, inlet velocity, and solution to anti-solvent mass flow ratio. To this end, a CFD model featuring population balance, multi-environment micro-mixing, and energy equations is developed and validated against experimental data. Then, the effects of the aforementioned parameters on the main performance indices such as crystal density, mean diameter, and product mass flow rate are investigated through the CFD model.

The rest of the paper is organized as follows. The CFD model is detailed is Section "Model Equations". The solution procedure including how the CFD model is set up and solved is discussed in “Numerical Solution Procedure” section. The simulation results are presented in “Results and Discussion” section, which is followed by concluding remarks in “Conclusions” section.

## Model equations

The model is composed of the PBEs and the equations for micro-mixing, linear momentum conservation, energy conservation, and crystallization kinetics. The model is able to predict the crystal size distribution (CSD) and the probability density function (PDF) describing local fluctuations in the turbulent flow field^24^. Non-ideal solubility was considered by $\Delta {H}_{mix}\ne 0$, and PBEs was coupled with the equations of mass transfer, momentum and energy^25^. Macro mixing was governed by the transfer equations and the k-ε turbulence model^26^. The crystal size distribution is numerically modeled using a High-Resolution, Finite-Volume, Semi-discrete Central Schemes and micro mixing with a hypothetical multi-environment PDF model^24–27^. Details of the model are described in the following sections.

### Population balance equation

The crystal population balance can be modeled as follows (see e.g.,^19^).

1 $$\frac{\partial f}{{\partial t}} + \mathop \sum \limits_{i} \frac{{\partial \left[ {G_{i} \left( {r_{i} .c.T} \right)f} \right]}}{{\partial r_{i} }} + \nabla \cdot \left( {\vec{v}f - D_{t} \nabla \cdot f} \right) = B\left( {f.c.T} \right)\Pi \delta \left( {r_{i} - r_{i0} } \right) + h\left( {f.c.T} \right),$$

where the particle number density function,* f* is a function of spatial coordinates (x, y, z), internal coordinates (*r*_*i*_), and time (*t*). Equation (1) assumes that the particles follow the stream lines in the flow field^28^. This assumption is a good approximation for organic pharmaceutical crystals, whose density is close to the liquid phase and their size is in micron range^28,29^. Based on this fact, breakage of particles due to particle collision is negligible and can be ignored. The population balance equation for the size of crystals (i.e., classes) is defined continuously. However, this would create an infinite number of equations and crystal sizes in the simulation, which cannot be handled in practice. Therefore, the PBE must be discretized to a finite number of sizes. The method high-resolution, finite-volume, semi-discrete central scheme^26^ was used to discretize the population balance equation. This method yields high accuracy simulations and eliminates non-physical fluctuations that can otherwise occur with classical methods^30^. This method has at least the second-order accuracy^30,31^.

The calculations are performed on the interval $\Delta x = x_{{j + \frac{1}{2}}} - x_{{j - \frac{1}{2}}}$, which is the length of class. The second-order semi-discrete method is as follows^30,31^:

2 $$\frac{{\text{d}}}{{{\text{d}}t}}\,f_{j} \left( t \right) = - \frac{{H_{{j + \frac{1}{2}}} \left( t \right) - H_{{j - \frac{1}{2}}} \left( t \right)}}{{\Delta x_{j} }}$$

*f*_*j*_ is the average number density of crystals of size *j* in cell and *H* is the flux transit through the boundary areas of the cell. For crystal growth, the numerical flux is as follows^29,31^.

3 $$H_{{j + \frac{1}{2}}} \left( t \right) = \frac{{u\left( {f_{{j + \frac{1}{2}}}^{ + } \left( t \right)} \right) + u\left( {f_{{j + \frac{1}{2}}}^{ - } \left( t \right)} \right)}}{2} - \frac{{G_{{j + \frac{1}{2}}} }}{2}\left[ {f_{{j + \frac{1}{2}}}^{ + } \left( t \right) - f_{{j + \frac{1}{2}}}^{ - } \left( t \right)} \right]$$

4 $$H_{{j - \frac{1}{2}}} \left( t \right) = \frac{{u\left( {f_{{j - \frac{1}{2}}}^{ + } \left( t \right)} \right) + u\left( {f_{{j - \frac{1}{2}}}^{ - } \left( t \right)} \right)}}{2} - \frac{{G_{{j - \frac{1}{2}}} }}{2}\left[ {f_{{j - \frac{1}{2}}}^{ + } \left( t \right) - f_{{j - \frac{1}{2}}}^{ - } \left( t \right)} \right]{ }$$

where $G_{j \pm 1/2}$ is the growth rate *j* of the crystal size (class *j* ‌). The dissolution flux is:

5 $$H_{{j - \frac{1}{2}}} \left( t \right) = \frac{{u\left( {f_{{j - \frac{1}{2}}}^{ + } \left( t \right)} \right) + u\left( {f_{{j - \frac{1}{2}}}^{ - } \left( t \right)} \right)}}{2} + \frac{{G_{{j - \frac{1}{2}}} }}{2}\left[ {f_{{j - \frac{1}{2}}}^{ + } \left( t \right) - f_{{j - \frac{1}{2}}}^{ - } \left( t \right)} \right]$$

6 $$H_{{j + \frac{1}{2}}} \left( t \right) = \frac{{u\left( {f_{{j + \frac{1}{2}}}^{ + } \left( t \right)} \right) + u\left( {f_{{j + \frac{1}{2}}}^{ - } \left( t \right)} \right)}}{2} + \frac{{G_{{j + \frac{1}{2}}} }}{2}\left[ {f_{{j + \frac{1}{2}}}^{ + } \left( t \right) - f_{{j + \frac{1}{2}}}^{ - } \left( t \right)} \right]$$

And the average numerical values are given as follows^31^:

7 $$u_{{j + \frac{1}{2}}}^{ + } \left( t \right) = u_{j + 1} \left( t \right) - \frac{\Delta x}{2}\left( {u_{x} } \right)_{j + 1} \left( t \right)$$

8 $$u_{{j + \frac{1}{2}}}^{ - } \left( t \right) = u_{j} \left( t \right) + \frac{\Delta x}{2}\left( {u_{x} } \right)_{j} \left( t \right)$$

the derivatives are estimated by the minmod limiter^26,31^:

9 $$\left( {u_{x} } \right)_{j}^{n} = minmod\left\{ {\begin{array}{*{20}c} {\theta \frac{{ u_{j}^{n} - u_{j - 1}^{n} }}{\Delta x}} \\ \end{array} , \frac{{u_{j + 1}^{n} - u_{j - 1}^{n} }}{2\Delta x}, \theta \frac{{u_{j + 1}^{n} - u_{j}^{n} }}{\Delta x}} \right\},\quad 1 \le \theta \le 2$$

where the minmode is defined as follows:

10 $$minmod\;\alpha_{1} , \alpha_{2} , \ldots = \left( {min\left\{ {\alpha_{i} } \right\}\quad if\quad \alpha_{i} > 0,\;max\left\{ {\alpha_{i} } \right\}\quad if\quad \alpha_{i} < 0,\quad 0\quad otherwise} \right)$$

The value *θ* = 1.5 is usually chosen to minimize the amount of scatter relative to non-physical smoothing by minimizing non-physical fluctuations^26^. More details about these limiters can be found in^26,30,32^. Returning to Eq. (2), the following semi-discrete PBEs are obtained after integration with respect to *r* in each cell, substituting Eqs. (3)–(6) and simplifying:

11 $$\frac{{\text{d}}}{{{\text{d}}t}}f_{{{j}}} \left( t \right) = - \frac{1}{\Delta r}G_{{j + \frac{1}{2}}} \left[ {f_{j} \left( t \right) + \frac{\Delta r}{2}\left( {f_{r} } \right)_{j} \left( t \right)} \right] - G_{{j - \frac{1}{2}}} \left[ {f_{j - 1} \left( t \right) + \frac{\Delta r}{2}\left( {f_{r} } \right)_{j - 1} \left( t \right)} \right]$$

$$if{ }\Delta C > 0$$

12 $$\frac{{\text{d}}}{{{\text{d}}t}}f_{{{j}}} \left( t \right) = - \frac{1}{\Delta r}G_{{j + \frac{1}{2}}} \left[ {f_{j + 1} \left( t \right) + \frac{\Delta r}{2}\left( {f_{r} } \right)_{j + 1} \left( t \right)} \right] - G_{{j - \frac{1}{2}}} \left[ {f_{j} \left( t \right) + \frac{\Delta r}{2}\left( {f_{r} } \right)_{j} \left( t \right)} \right]$$

*if* $$\Delta C < 0$$.

where *f*_*j*_ is the average cell number density approximated by Eq. (2) and derivatives (*f*_*r*_)_*j*_ (by the minmod limiter^25^. To solve the semi-discrete PBE using CFD, Eqs. (11) & (12) are recast on a mass basis. The overall mass balance of the system is satisfied by combining this equation with the solute, solvent, and anti-solvent equations^19^:

13 $$f_{w.j} = \rho_{c} k_{v} \mathop \smallint \limits_{{r_{j - 1/2} }}^{{r_{j + 1/2} }} r^{3} f_{j} {\text{d}}r = \frac{{\rho_{c} k_{v} f_{j} }}{4\Delta r}\left[ {\left( {r_{{j + \frac{1}{2}}} } \right)^{4} - \left( {r_{{j - \frac{1}{2}}} } \right)^{4} } \right]$$

Finally, the semi-discrete PBE equations on the mass basis are:

14 $$\frac{{\text{d}}}{{{\text{d}}t}}\,f_{w.j} \left( t \right) + \nabla \cdot \left( {\vec{V}f_{w.j} - D_{t} \nabla \cdot f_{w.j} } \right) =$$

$$\frac{{\rho_{j} k_{v} }}{4\Delta r}\left[ {\left( {r_{{j + \frac{1}{2}}} } \right)^{4} - \left( {r_{{j - \frac{1}{2}}} } \right)^{4} } \right] \times \left\{ { - G_{{j + \frac{1}{2}}} \left[ {f_{j} + \frac{\Delta r}{2}\left( {f_{r} } \right)_{j} } \right] + G_{{j - \frac{1}{2}}} \left[ {f_{j - 1} + \frac{\Delta r}{2}\left( {f_{r} } \right)_{j - 1} } \right] + B} \right\}$$

*if* $$\Delta C > 0$$

15 $$\frac{{\text{d}}}{{{\text{d}}t}}\,f_{w.j} \left( t \right) + \nabla \cdot \left( {\vec{V}f_{w.j} - D_{t} \nabla \cdot f_{w.j} } \right) =$$

$$\frac{{\rho_{c} k_{v} }}{4\Delta r}\left[ {\left( {r_{{j + \frac{1}{2}}} } \right)^{4} - \left( {r_{{j - \frac{1}{2}}} } \right)^{4} } \right] \times \left\{ { - G_{{j + \frac{1}{2}}} \left[ {f_{j + 1} + \frac{\Delta r}{2}\left( {f_{r} } \right)_{j + 1} } \right] + G_{{j - \frac{1}{2}}} \left[ {f_{j} + \frac{\Delta r}{2}\left( {f_{r} } \right)_{j} } \right]} \right\}\;if\;\Delta C < 0$$

where $f_{w.j}$ is the cell-averaged crystal mass and has the units, kg/m^3^, $\rho_{c}$ is the crystal density, $k_{v}$ is the volume factor, and $\left( {f_{r} } \right)_{j}$ is an approximate derivative estimated by the minmod limiter.

### Multi-environment PDF model (micro-mixing model equations)

A multi-environment model of micromixing (PDF) taken from^21^ was used to model the mixing effects^24^. In this method, each CFD cell is divided into three probability states or environments: (1) solution environment that contains the mixture of methanol and lovastatin, (2) water environment as the anti-solvent, and (3) a combination of the two that represents the mixed state. The PDF is discretized into a finite set of δ functions^21^:

16 $$f_{\phi } \left( {\psi .x.t} \right) = \mathop \sum \limits_{n = 1}^{3} P_{n} \left( {x.t} \right)\mathop \prod \limits_{\alpha = 1}^{Ns} \delta \left[ {\psi_{\alpha } - \phi_{\alpha n} \left( {x.t} \right)} \right],$$

in which $f_{\Phi }$ is a common PDF of all scalars and *N*_*s*_ is the total number of species, *P*_*n*_ is probability of the environment *n*, and $\phi_{\alpha }$_*n*_ is the average composition of species *α* in the environment *n*. The weighted concentration in each environment is as follows:

17 $$s_{n} \equiv P_{n} \Phi_{n}$$

The following equations describe the transport of probability and transport of species in each environment *n*: 

18 $$\frac{\partial P}{{\partial t}} + \nabla \cdot \left( {\vec{v}P - D_{t} \nabla \cdot P} \right) = G\left( P \right) + G_{s} \left( P \right)$$

19 $$\begin{aligned} & \frac{{\partial s_{n} }}{{\partial t}} + \nabla \cdot \left( {\vec{v}s_{n} - D_{t} \nabla \cdot s_{n} } \right) \\ & = M^{n} \left( {p \cdot s_{1} \ldots s_{{N_{e} }} } \right) + M_{s}^{n} \left( {p \cdot s_{1} \ldots s_{{N_{e} }} } \right) + P_{n} S\left( {\phi _{n} } \right) \\ \end{aligned}$$

where *G* is the rate of change of probability in each environment and *M*^*n*^ is the rate of change of $s_{n}$ due to micro mixing, and *S* is the chemical source term. *G*_*s*_ and $M_{s}^{n}$ are added to these equations to remove the effect of false dissipation rate in the transport equations^33^. The following equations should also be held due to the conservation of probability:

20 $$\mathop \sum \limits_{n = 1}^{N} P_{n} = 1$$

21 $$\mathop \sum \limits_{n = 1}^{N} G_{n} \left( P \right) = 0$$

The averages remain unchanged during micro-mixing which results in:

22 $$\mathop \sum \limits_{n = 1}^{N} M^{n} \left( {P,s_{1} , \ldots ,s_{{N_{e} }} } \right) = 0$$

The average composition of the species is as follows:

23 $$\Phi = \mathop \sum \limits_{n = 1}^{N} P_{n} \Phi_{n} = \mathop \sum \limits_{n = 1}^{N} s_{n}$$

The advantage of using a multi-environment PDF model over other micro-mixing models is that it can be easily incorporated into existing CFD codes, where the transfer equations, Eqs. (18) and (20), can be solved similar to other transport equations. Since the compounds in the environments 1 and 2 are known based on the initial conditions and the feed, Eq. (18) applies only to all species in environment 3 where all species are mixed and crystallization occurs. It is also assumed that the crystal particles follow the fluid flow streamlines in the crystallizer because the particles in these systems are very small^28,29^. Equation (19) is used to evaluate the mixing fraction in Environment 3. ${\langle \xi \rangle }_{3}$ is the relative fraction of fluids in environments 1 and 2, ${\langle \xi \rangle }_{1}$ is mixing fraction in environment 1 (equal to 1) and ${\langle \xi \rangle }_{2}$ is the mixing fraction in environment 2 (equal to 0). If ${\langle \xi \rangle }_{3}$ is equal to 0.5, it means that there is an equal fraction of both fluids in the mixing environment. Table 1 lists the relations that are used to evaluate micro-mixing terms.

**Table 1:** The relations for evaluating micro-mixing terms^20^.

Model variables	${M}^{n} G$	${G}_{s} {M}_{s}^{n}$
${P}_{1}$	$-\gamma {P}_{1}\left(1-{P}_{1}\right)$	${\gamma }_{s}{P}_{3}$
${P}_{2}$	$-\gamma {P}_{2}\left(1-{P}_{2}\right)$	${\gamma }_{s}{P}_{3}$
${P}_{3}$	$\gamma {[P}_{1}\left(1-{P}_{1}\right)+{P}_{2}\left(1-{P}_{2}\right)]$	${-2\gamma }_{s}{P}_{3}$
${\langle s\rangle }_{3}$	$\gamma {[P}_{1}\left(1-{P}_{1}\right){\langle \Phi \rangle }_{1}+{P}_{2}\left(1-{P}_{2}\right){\langle \Phi \rangle }_{2}]$	$-{\gamma }_{s}{P}_{3}({\langle \Phi \rangle }_{1}+{\langle \Phi \rangle }_{2})$
	$$\gamma =\frac{{\varepsilon }_{\xi }}{{P}_{1}\left(1-{P}_{1}\right)\left(1-{\langle \xi \rangle }_{3}^{2}\right)+{P}_{2}\left(1-{P}_{2}\right){\langle \xi \rangle }_{3}^{2}}$$	
	$${\gamma }_{s}=\frac{2{D}_{t}}{{\left(1-{\langle \xi \rangle }_{3}\right)}^{2}+{\langle \xi \rangle }_{3}^{2}}\frac{\partial {\langle \xi \rangle }_{3}}{\partial x}\frac{\partial {\langle \xi \rangle }_{3}}{\partial x}$$	
	$$\langle {{\xi }{\prime}}^{2}\rangle ={P}_{1}\left(1-{P}_{1}\right)-2{P}_{1}{P}_{3}{\langle \xi \rangle }_{3}+{P}_{3}\left(1-{P}_{3}\right){\langle \xi \rangle }_{3}^{2}$$	

In Table 1, ${\langle \phi \rangle }_{n}$ is evaluated using Eq. (23). The value of P3 can also be determined from Eq. (22), (*P*_*3*_ = *P*_*1*_ + *P*_*2*_). For a fully developed scalar spectrum, the scalar loss or dispersion rate, $\upvarepsilon _{\xi }$ is as follows.

24 $$\varepsilon_{\xi } = C_{\Phi } \xi^{{\prime}{2}} \frac{\varepsilon }{k}$$

where the dispersion constants,$C_{\Phi } = 2$, *k*, and *ε* are turbulent kinetic energy and kinetic dissipation rates, respectively^34^.

### Conservation of energy

It is assumed that the three environments are in thermal equilibrium in each cell^24^. This means that a single energy equation is considered for all the three environments. The general form of the energy equation for an incompressible flow can be written as follows:

25 $$\frac{\partial }{\partial t}\left( {\rho E} \right) + \nabla \cdot \left[ {\vec{v}\left( {\rho E + P} \right)} \right] = \nabla \left[ {K_{eff} \nabla T + \left( {\overline{\tau }_{eff} \cdot \vec{v}} \right)} \right] + S_{h}$$

*S*_*h*_ is heat source due to crystallization and mixing between solvent and anti-solvent:

26 $$S_{h} = S_{3} \left( { - \Delta H_{mix} } \right) + \left( {\Sigma_{j} S_{{f_{w.j} }} } \right)\left( { - \Delta H_{crys} } \right)$$

where *S*_*3*_ $(M^{n} + M_{s}^{n} )$ is the rate of increase in solvent concentration and anti-solvent in environment 3. $\Sigma_{j} S_{{f_{w.j} }}$ is the rate of increase in total crystal mass in the environment 3 )$S_{{f_{w.j} }}$ is the mass density of a crystal in class *j.*( $\Delta H_{mix}$ is the heat of mixing of solvent with anti-solvent^35^ and $\Delta H_{crys}$ is the crystallization heat of drug^36^.

### Crystallization of lovastatin

The solubility of lovastatin can be obtained as follows^21^.

27 $$C^{*} \left( {\frac{kg}{{kg of solvents}}} \right) = 0.001\exp \left( {15.45763\left( {1 - \frac{1}{\theta }} \right)} \right)*$$

$$\left\{ {\begin{array}{*{20}l} {( - 2.7455{*}10^{ - 4} w_{as}^{3} + 3.3716{*}10^{ - 2} w_{as}^{2} - 1.6704{\text{w}}_{{{\text{as}}}} + 33.089)} \hfill & {{\text{for}}\quad {\text{w}}_{{{\text{as}}}} \le 45.67} \hfill \\ {\left( {1.7884{\text{*w}}_{{{\text{as}}}} + 1.7888} \right)} \hfill & {{\text{for}}\quad {\text{w}}_{{{\text{as}}}} \le 45.67} \hfill \\ \end{array} } \right\}$$

$$\theta = \frac{T}{{T_{ref} }},\quad T_{ref} = 296K$$

and the growth and nucleation rates are as follows^37^.

28 $$B = B_{hemogeneous} + B_{hetrogeneous}$$

29 $$B_{hemogeneous} \;at\;23^\circ {\text{C}}\left( {\frac{1}{{S.m^{3} }}} \right) = 6.97*10^{14} exp\left( { - \frac{15.8}{{\left[ {\ln S} \right]^{2} }}} \right)$$

30 $$B_{hetrogeneous} \;at\;23^\circ {\text{C}}\left( {\frac{1}{{S.m^{3} }}} \right) = 2.18*10^{8} exp\left( { - \frac{0.994}{{\left[ {\ln S} \right]^{2} }}} \right)$$

31 $$G\;at\;23^\circ {\text{C}}\left( \frac{m}{S} \right) = 8.33*10^{ - 30 } \left[ {2.46*10^{3 } *\ln S} \right]^{6.7}$$

where *w*_*as*_ is the weight percent of the anti-solvent (water) and $S = \frac{C}{{C^{*} }}$ is the supersaturation ratio (*c* and c* are the solution and saturated concentrations, respectively).

## Numerical solution procedure

The details of the CFD model including the geometry, meshing, and boundary conditions as well as the numerical tool are presented in this section.

### Computational domain

**Figure 1:**
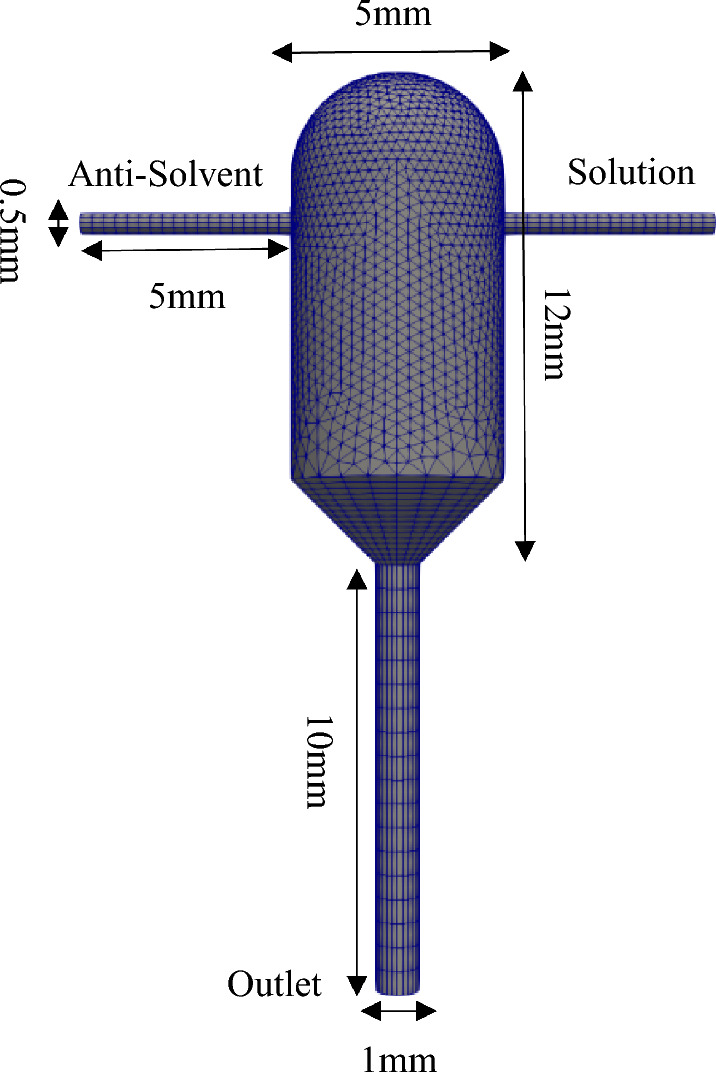
Geometric specifications and the mesh created for the jet crystallizer.

Based on the experimental work in^11^, a three-dimensional geometry was created, as shown in Fig. 1. For meshing a combination of hexagonal and tetrahedron cells was used to fill the computation space and get a quality mesh. The mesh density around the jet outlets and in the region where jets collide is higher than other parts. Liquid enters the crystallizer through two inlets and exit it from the outlet.

### Numerical solution method for simulation

The model equations were implemented in the OpenFOAM software using in the C +  + programming language. The population balance equation was divided into 30 classes with *∆r* = *2* $\mu m.$ The coupling between pressure and velocity equations were performed using pimple algorithm, which combines the SIMPLE algorithm as a method of estimating pressure and the PISO algorithm to modify the second pressure correction and explicitly modify the velocity and pressure. The discretization methods for convection and diffusion terms (Laplacian) were bounded second-order linear upwind and unbounded second-order linear limited, respectively. All the simulations continued until they reach steady state.

There are four boundaries in the domain that should be defined for each field in the simulation: anti-solvent inlet, solution inlet, walls, and outlet. For the inlets, the velocity was set to a fixed value (based on the conditions of simulation). A no-slip condition was used for the walls, and a fully developed condition was set for the outlet (pressureInletOutletVelocity). The temperature and all concentrations were set to fixed values for the inlets, zero gradient (zeroGradient) for the (heat and mass insulated) wall, and fully developed condition for the outlet (inletOutlet). The value of pressure was set to a fixed value in the outlet and zero gradient for the other boundaries. Regarding the turbulent fields, fully developed condition was considered for the outlet and turbulent wall function for the wall. The initial conditions were set as follows. The initial velocity, (gauge) pressure, concentration of crystals, and turbulent fields were all set to zero. The initial temperature was considered to be 25 °C.

### Operating conditions

The simulations were performed for lovastatin-methanol (as solute–solvent) solution fed through the feed inlet and the anti-solvent (pure water) fed through another inlet at 25 °C. The density of water, methanol and lovastatin is 997.1, 782, 1273 kg/m^3^, respectively. The kinematic viscosities of water and methanol are 8.976 × 10^–7^, 6.87 × 10^–7^ m^2^/s respectively.

## Results and discussion

In this section, the CFD model is first validated against experimental data from the literature. Then, effects of key process parameters on the crystallization are investigated and discussed: changes in the inlet flow velocity, lovastatin inlet supersaturation ratio, and solution to anti-solvent mass flow ratio on the density, mean diameter, and mass rate of crystals leaving the crystallizer.

### Model validation

**Figure 2:**
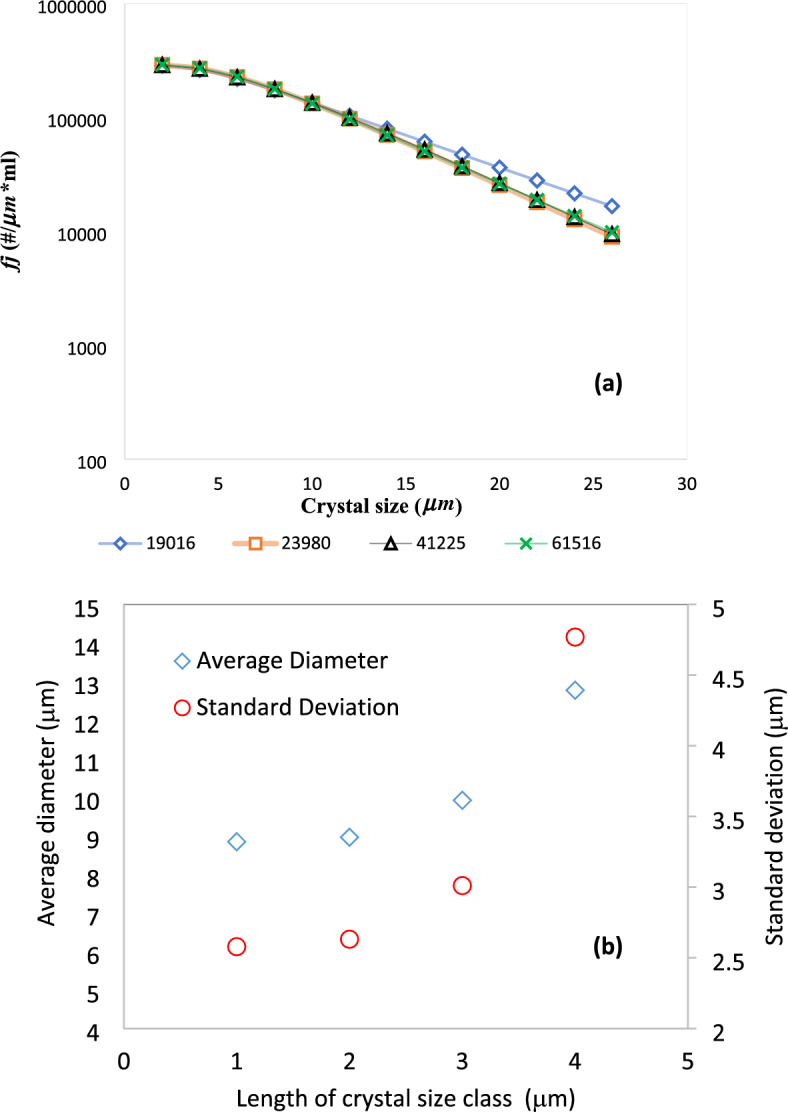
Discretization-independency results, (**a**) mesh independency for the number density based on the number of cells used in the simulations and (**b**) independency of the simulation results of the length of crystal size class.

A mesh independency test is first performed to ensure the independency of the numerical results from the discretization resolution. The results of the mesh independency analysis are shown in Fig. 2a. Since the particle size distribution is the most important parameter in this work, *f*_j_ (number density) is chosen as the basis for the mesh independence analysis. Increasing the number of mesh cells from 19,019 to 23,980 elements has a significant effect on the number density. However, further increases in the number of mesh elements has a negligible effect on the result. Therefore, all simulations in the next sections are performed with the mesh with 23,980 cells.

Another factor that may affect the simulation results is the length of the crystal size class. A uniform classification was used for representing crystal sizes over a size span of 0 to 60 μm. Cheng et al.^23^ showed that uniform classification of crystal size can well capture the CSD when the jet velocity is below 4 m/s. To investigate the impact of class discretization on the computed CSD, the length of the size class was varied from 4 μm (corresponding to 15 classes) to 1 μm (corresponding to 60 classes) and the mean and standard deviation of crystal size in the crystallizer outlet were compared. The results are depicted in Fig. 2b. As the length of size class decreases from 4 to 2 μm, both mean and standard deviation are significantly decreased. However, the change in the mean and the standard deviation is negligible going from 2 to 1 μm (1.5% for the mean and 2% for the standard deviation). Therefore, 2 μm is considered an optimal length for the size class that can save up to 50% computation time without sacrificing the accuracy, compared to 1 μm-length class. In all the simulations, the size span covered by crystal size classes is from 2 to 60 μm. This range is wide enough to hold all sizes of crystals that are produced in the simulations, as seen in the subsequent sections.

**Figure 3:**
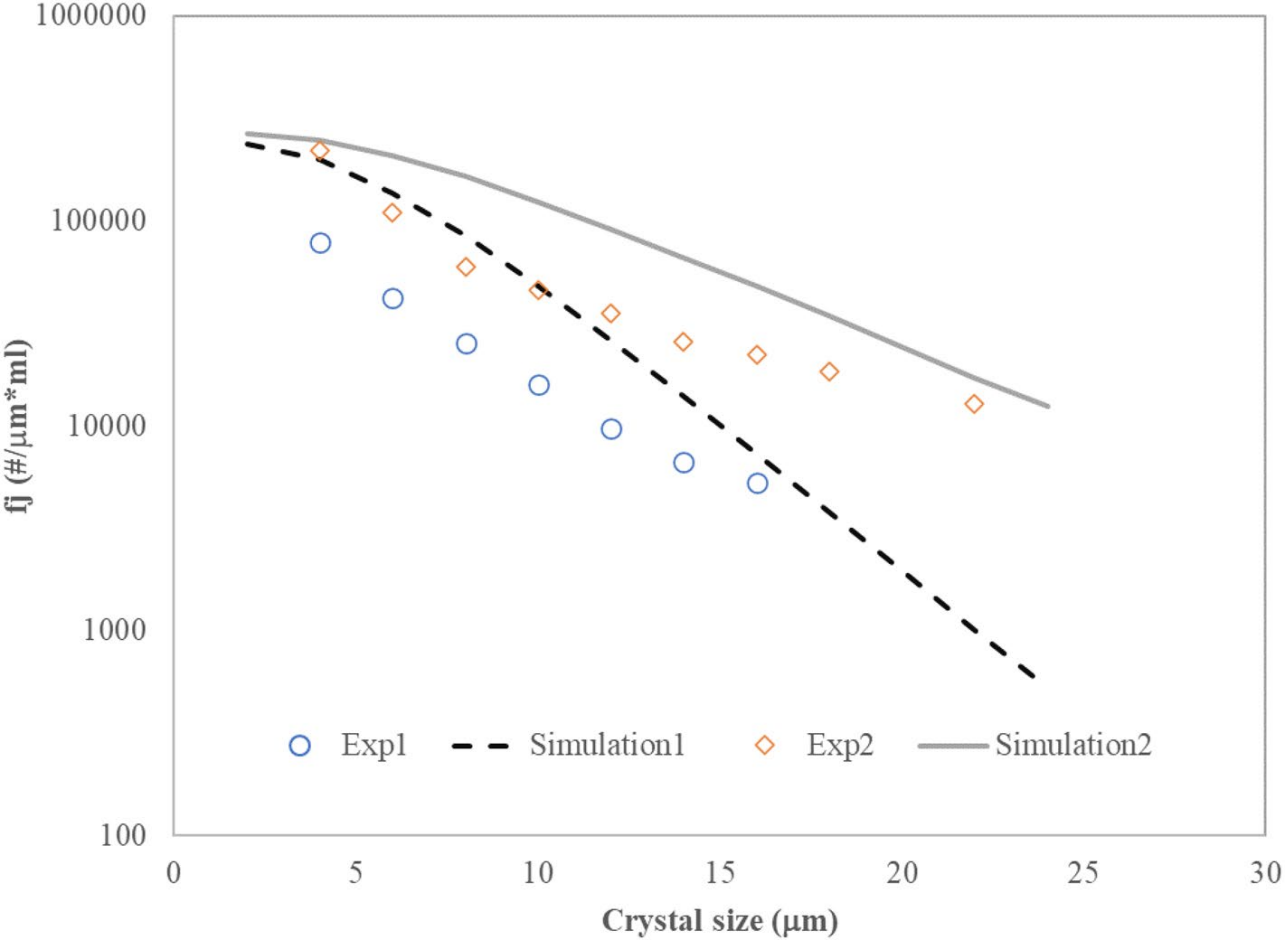
Comparison of simulation results with experimental data at different inlet velocities. Exp1: solution velocity is 1.17 m/s, anti-solvent velocity is 1.03 m/s—Exp2: solution velocity is 1.97 m/s, anti-solvent velocity is 1.74 m/s^11^.

With the mesh independency established, a set of simulations based on the experimental work in^11^ are performed for model validation. Figure 3 compares the outlet crystal size distribution from the simulation with the experimental data. The model follows the decreasing trends of the number density with crystal size and can correctly reflect the effect of jet velocity on the size distribution and mean crystal size. In the experiments, the particle size distribution decreases with increasing the inlet velocity (due to the decreased residence time), which is also observed in the simulation results. At both inlet velocities, however, the model overpredicts the number density. The differences between the simulation results and experimental data may be related to the assumptions made in the model, such as neglecting crystal aggregation and breakage and the adherence of drug particles to flow lines^29,30^. Another source of this overprediction can be the uncertainty in the nucleation rate of crystals (Eq. (31)). If Eq. (31) overpredicts the actual nucleation rate, the resulting crystal number distribution would be higher, as we see in Fig. 3.

### Numerical case studies

A number of case studies are presented in following subsections in order to investigate the effect of some of the main operating conditions on the crystallization performance in steady-state conditions. The inlet solution and anti-solvent streams enter the crystallizer at 25 °C (298 K) in all the simulations. In all the crystallizer figures shown subsequently, the left and the right inlets correspond to the anti-solvent and the solution streams, respectively.

#### Effect of inlet supersaturation ratio

Here, the simulations are performed at different supersaturation ratios of the solution stream (i.e., 8.8, 7.8, and 6.8), but the same velocity of 1.5 m/s for both inlet streams. As seen in Table 2, the inlet supersaturation ratio has a direct effect on the growth and nucleation rates, with a higher supersaturation ratio resulting in increased growth and nucleation rates in the crystallizer. Also, the temperature inside the crystallizer increases to 35 °C in all the three simulations. This is mainly due to the heat of mixing as the crystallization heat of lovastatin is too small to have a considerable contribution to temperature change^21^.

**Table 2:** Main results of the CFD simulations at different supersaturation ratios.

Inlet supersaturation ratio	Growth rate (m/s) × 10–5	Nucleation rate #/(m3 × s)) × 10 + 12	Average crystals diameter $(\mu m)$	Standard deviation $(\mu m)$	Crystals outlet flow rate (mg/s)
6.8	1.36	2.53	6.75	1.68	7.21 × 10^–2^
7.8	1.79	3.72	8.89	2.56	1.51 × 10^–1^
8.8	1.92	4.22	11.64	6.87	4.34 × 10^–1^

**Figure 4:**
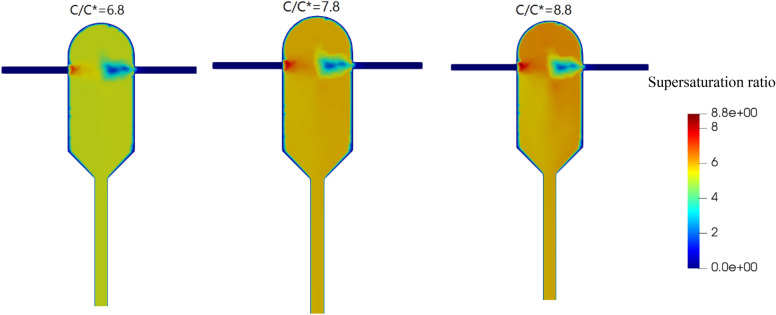
Supersaturation ratio field at supersaturation ratios of 8.8, 7.8, 6.8 and the same inlet velocities (1.5 m/s).

Mixing the solution with the anti-solvent reduces solubility, creating supersaturation for crystallization. As shown in Fig. 4, when the saturation of the inlet solution is higher, a higher supersaturation ratio occurs inside the crystallizer. With increasing the inlet supersaturation from 6.8 to 7.8, the average supersaturation ratio in the crystallizer increases by 11%. However, the rate of increase goes down with higher inlet supersaturation ratios, as the average supersaturation ratio in the crystallizer increases by only 0.3% when the inlet supersaturation ratio is increased from 7.8 to 8.8.

**Figure 5:**
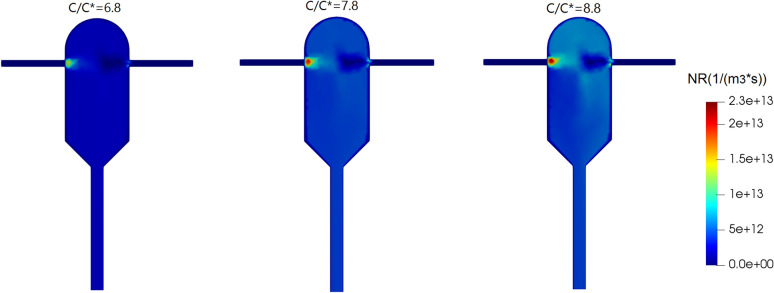
Nucleation rate change field at different supersaturation ratios and the same inlet velocities (1.5 m/s).

The field of changes in the nucleation rate at different inlet supersaturation ratios is shown in Fig. 5. As seen in Fig. 5, at the point of mixing the anti-solvent with the solution, the highest nucleation rates occur. The field of nucleation rate can be explained by the field of supersaturation ratio shown in Fig. 4 and the fact that supersaturation has a direct impact on the nucleation rate (see Eqs. (28–30)). The growth rate field follows a similar pattern and is omitted for brevity. It is also observed that at higher inlet supersaturation ratios, nucleation rates are higher. By increasing the inlet supersaturation from 6.8 to 7.8, the nucleation rate and the growth rate increase by 47% and 31%, respectively. Also, by increasing the inlet supersaturation ratio from 7.8 to 8.8, the nucleation rate increases by 13.44% and the growth rate by 7.26%, respectively. Therefore, the sensitivity of the nucleation and growth rates goes down at higher inlet supersaturation ratios.

**Figure 6:**
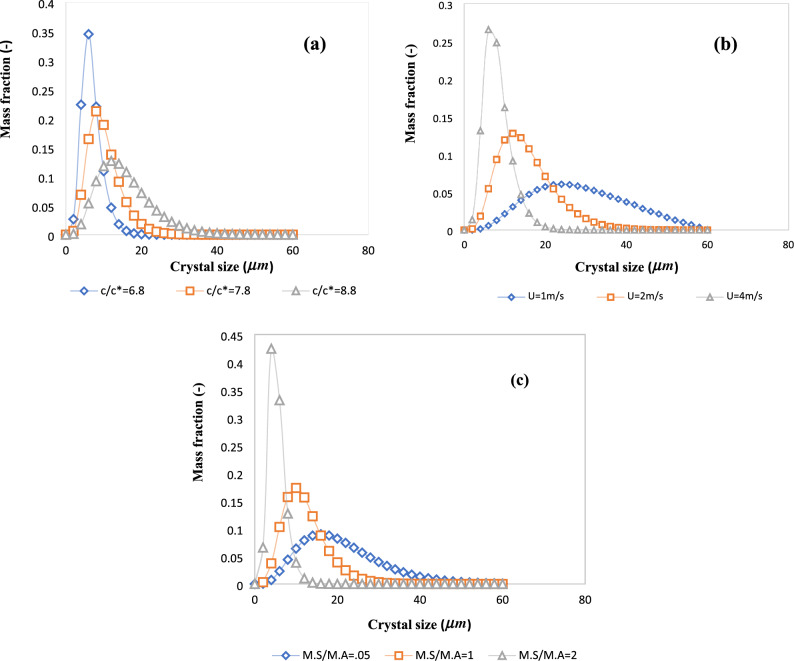
Mass distribution of crystals in the crystallizer outlet (**a**) at different inlet supersaturation ratios (in “Effect of inlet supersaturation ratio” section), (**b**) at different inlet velocities (“Effect of inlet velocity” section), and (**c**) at different inlet mass flow ratios (“Effect of solution to anti-solvent mass flow ratio” section).

Figure 6a shows the mass fraction distribution in the crystallizer outlet at different inlet supersaturation ratios. It is seen that the mass fraction distribution is narrower and the mean crystal size is smaller with a lower inlet supersaturation ratio. This is explained by the fact that the growth and nucleation rates are lower at lower supersaturation ratios (see Fig. 5). The key simulation results with different inlet supersaturation ratios are reported in Table 2. Interestingly, the crystals outlet flow rate increases by an order of magnitude and the average crystals diameter almost doubles when the inlet supersaturation ratio increases by only two units. However, a wider crystal size distribution could be a disadvantage of the higher inlet supersaturation ratio.

#### Effect of inlet velocity

**Figure 7:**
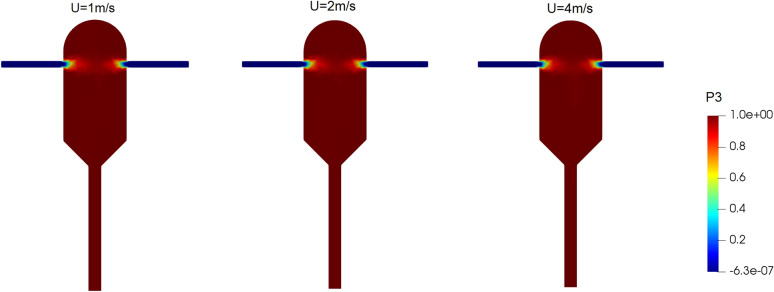
Volume fraction of the mixing environment (P3) at different inlet velocities of solution and anti-solvent streams It changes the nucleation rate of the field at different solute inlet velocities and anti-solvent currents in the supersaturated ratio of the input solution equal to 8.8.

In this case study, three simulations are run to investigate the effect of inlet velocity on the crystallization. From the first to the third simulation, velocities of 1, 2, and 4 m/s are set for both inlet streams (i.e., the same velocity for both inlets in each case). To observe the effects of speed on the particle mass distribution, the supersaturation ratio of the inlet solution was considered equal to 8.8 in all simulations. An increase in the inlet velocity is expected to increase the turbulence and yield better mixing^26^. Figure 7 shows the volume fraction of the mixing environment in the crystallizer for different inlet velocities. This quantity being closer to one indicates a better mixing of solution and anti-solvent in the crystallizer. As seen in Fig. 7, a very good mixing is achieved for all the three cases.

**Figure 8:**
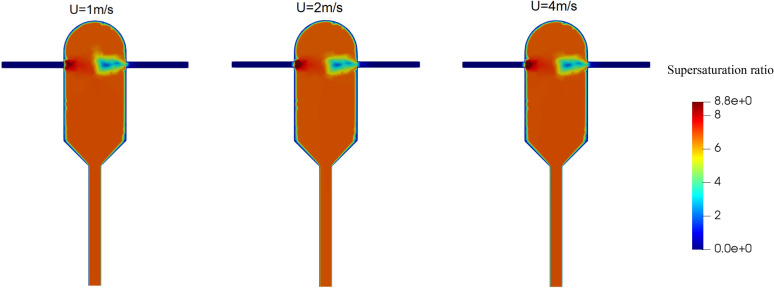
Field of supersaturation ratio at different inlet velocities of solution and anti-solvent streams in the supersaturated ratio of the input solution equal to 8.8.

Figure 8 shows the field of supersaturation ratio at different inlet velocities. It is seen that except in the area close to the solution inlet nozzle, an almost uniform supersaturation ratio occurs in the crystallizer. However, a closer look reveals that the supersaturation ratio is slightly higher at the anti-solvent inlet nozzle.

**Figure 9:**
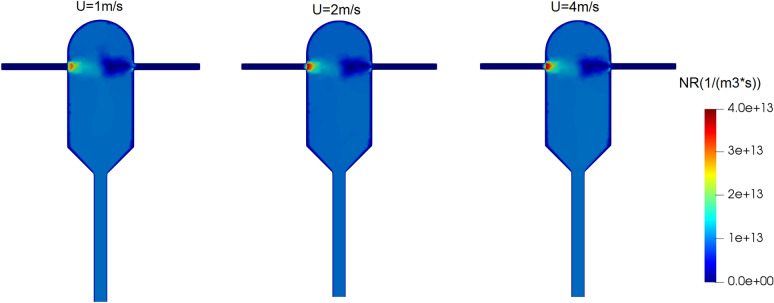
Nucleation rate changes field at different inlet velocities of solution and anti-solvent streams in the supersaturated ratio of the input solution equal to 8.8.

The fields of changes in the nucleation rate at different inlet velocities are shown in Fig. 9, where it is observed that the nucleation rate is almost the same in all the three cases. This can be justified from Fig. 8, where the fields of supersaturation ratio are also almost unchanged across different inlet velocities due to the sufficiently high mixing efficiency in all the cases. The fields of growth rate follow a similar pattern and is omitted for brevity.

The outlet mass fraction distribution of crystals at different inlet velocities is shown in Fig. 6b. It is seen that at higher inlet velocities, the crystal size distribution is narrower and the mean crystal size is smaller. This is explained by the shorter residence time at higher inlet velocities, which provides less opportunity for the crystals to grow.

The key simulation results with different inlet velocities are reported in Table 3. It is observed that the crystals outlet flow rate decreases by two orders of magnitude and the average crystals diameter decreases to almost one-third when the inlet velocities are increased from 1 to 4 m/s. The substantial decrease in these quantities could also be predicted from Fig. 6b, as explained before. Also, the supersaturation ratio and temperature inside the crystallizer exhibit no sensitivity to the inlet velocities, perhaps because even the lowest velocity studied here is high enough to yield a complete mixing. We can conclude that the inlet velocity does not alter the mixing condition in the crystallizer and it can be adjusted to achieve the desired mean size and size distribution of produced crystals.

**Table 3:** Main results of the CFD simulations at different inlet velocities.

Inlet velocity (m/s)	Mixing environment volume fraction (*P3*)	Growth rate (m/s) × 10^–5^	Nucleation rate (#/(m^3^ × s)) × 10^+12^	Supersaturation ratio	Temperature (K)	Average crystals diameter $\left(\mathrm{\mu m}\right)$	Standard deviation $(\mathrm{\mu m})$	Crystals outlet flow rate (mg/s)
1	0.9981	3.5706	9.9818	6.8388	308.12	23.41	11.76	3.79
2	0.9991	3.5673	9.9771	6.8403	308.15	11.14	6.07	5.11 × 10^–1^
4	0.9999	3.5591	0.9695	6.8422	308.21	7.40	4.16	8.36 × 10^–2^

#### Effect of solution to anti-solvent mass flow ratio

**Figure 10:**
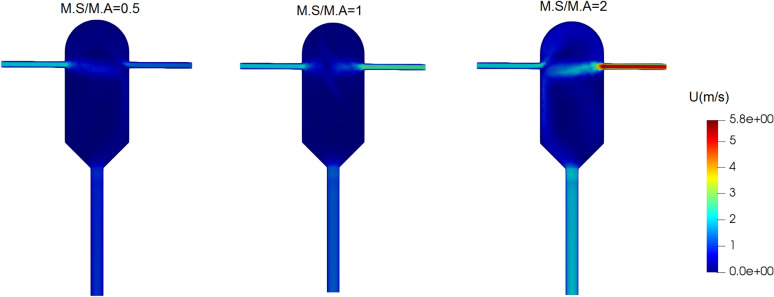
Velocity field inside the crystallizer at different mass flow ratios of solution to anti-solvent inlet streams in the supersaturated ratio of the input solution equal to 8.8.

In this section, the effect of the mass flow rate of the inlet solution (M.S) relative to that of the inlet anti-solvent (M.A) on the crystallization performance is investigated. The anti-solvent mass flow rate is fixed at 498.6 × 10^–6^ kg/s. The mass flow rate of the inlet solution is set to half, equal to, and twice the anti-solvent mass flow rate, resulting in different inlet mass flow ratios (M.S/M.A) in each simulation. To observe the effects of mass flow rate on particle size distribution, the supersaturation ratio of the inlet solution was considered equal to 8.8 in all simulations. The velocity field inside the crystallizer at different mass flow ratios is illustrated in Fig. 10. Interestingly, with the inlet mass flow ratio of M.S/M.A = 2, the anti-solvent stream is directed to the top of the crystallizer due to the high impact intensity and velocity of the solution stream.

**Figure 11:**
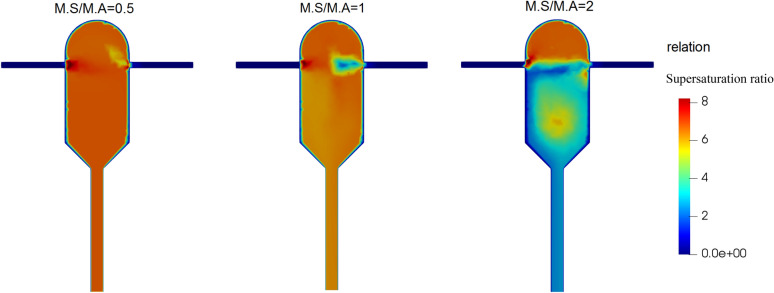
Supersaturation ratio changes field at different mass flow ratios of solution to anti-solvent inlet streams in the supersaturated ratio of the input solution equal to 8.8.

The supersaturation ratio field in the crystallizer at different mass flow ratios is depicted in Fig. 11. When the solution flow rate is half of the anti-solvent flow rate (i.e., the lowest mass flow ratio of M.S/M.A = 0.5), the supersaturation inside the crystallizer in the highest and the most uniform. This is because the anti-solvent content is enough to bring the mixture to a high supersaturation state. However, as the mass flow ratio is increased, the supersaturation decreases and starts to show non-uniformity. The lowest, most non-uniform supersaturation is observed for M.S/M.A = 2, where there is no enough anti-solvent for the mixture to yield satisfactory supersaturation.

**Figure 12:**
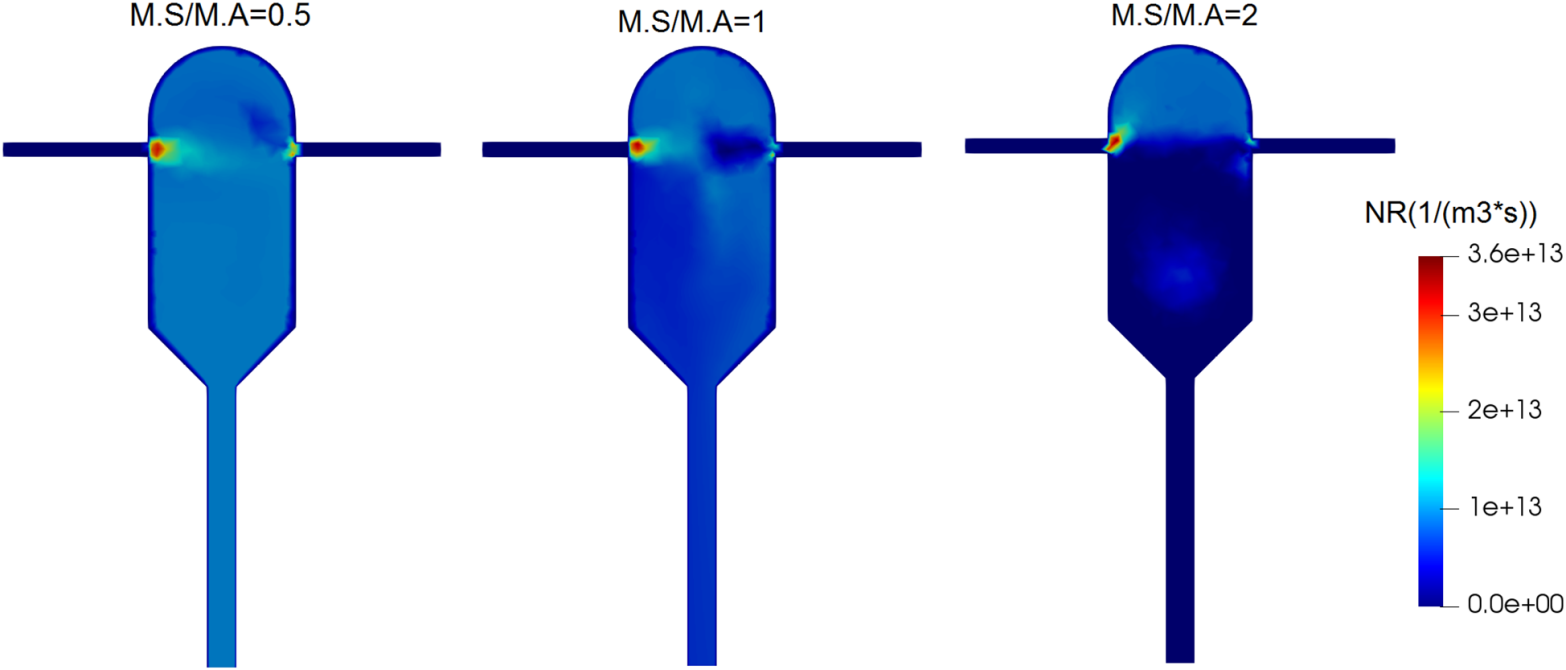
Nucleation rate changes field at different mass flow ratios of solution to anti-solvent inlet streams in the supersaturated ratio of the input solution equal to 8.8.

The field of changes in the nucleation rate at different inlet mass flow ratios is given in Fig. 12. In each case, the highest nucleation rate occurs to the anti-solvent nozzle. Also, the nucleation rate is highest and most uniform for M.S/M.A = 0.5, when the supersaturation is also highest and most uniform (Fig. 11). This is explained by the direct relationship between the two quantities. The growth rate exhibits a similar field and is omitted for brevity.

The outlet mass distributions of the crystals at different mass flow ratios are shown in Fig. 6c. It is observed that with a higher mass flow ratio, the crystal mass distribution is narrower and the mean crystal size is smaller. This is justified by the fact that less anti-solvent is being provided in case of a higher mass flow ratio, leading to less supersaturation and lower growth and nucleation rates.

The key simulation results with different mass flow ratios are provided in Table 4. Similar to the previous case studies, the temperature inside the crystallizer rises by 10 degrees mainly due to the heat of mixing. However, the temperature increase is insensitive to the mass flow ratio, perhaps because even the lowest mass flow ratio studied here is high enough to provide a complete mixing between the two inlet nozzles. Moreover, it is interesting to see that the mass flow ratio has a highly nonlinear impact on the growth and nucleation rates. In particular, the two quantities remain almost the same with M.S/M.A = 0.5 and M.S/M.A = 1. Nonetheless, they decrease by orders of magnitude when the mass flow ratio is further increased to M.S/M.A = 2.

**Table 4:** Main results of the CFD simulations at different inlet mass flow ratios.

M.S/M.A ratio	Growth rate (m/s) × 10^–5^	Nucleation rate (#/(m^3^ × s))	Temperature (K)	Average crystals diameter ($\mathrm{\mu m}$)	Standard deviation $(\mathrm{\mu m})$	Crystals outlet flow rate (mg/s)
0.5	3.43	9.51 × 10^+12^	308.33	13.43	8.75	1.06
1	3.35	9.27 × 10^+12^	308.47	9.65	5.88	2.52 × 10^–1^
2	0.021	6.73 × 10^+7^	308.80	5.66	2.72	6.68 × 10^–3^

## Conclusions

In this paper, the performance of a continuous jet crystallizer for lovastatin crystallization was studied using a CFD model augmented with population balance and micro mixing. It was found that the key operating parameters have a nonlinear, significant effect on the performance indices including the crystal size. In particular, the crystal production rate changes by orders of magnitude as a result of change to the inlet supersaturation, velocity, or mass flow ratios. It was also found that for larger crystals and higher production rate, the inlet supersaturation ratio should be increased, and the inlet velocities and M.S/M.A ratio should be kept low. Among the three parameters studied, the crystal mean size is the least sensitive to the inlet supersaturation ratio, and almost equally sensitive to the inlet velocities and M.S/M.A. The detailed computational results obtained in this work reveal useful insights for scaleup and design improvement of jet crystallizers for the production of lovastatin or other pharmaceutical drugs. The complexity of the process makes it impossible to predict its sensitivity to key operating parameters in a quantified manner without performing a high-fidelity simulation study. Also, the use of the open-source software OpenFOAM enables easy extension of the current model to include other crystallization phenomena such as aggregation and breakage.

### Supplementary Information

Supplementary Information 1.

Supplementary Information 2.

Supplementary Information 3.

## Data Availability

All the datasets used and/or analyzed during the current study available from the corresponding author on reasonable request. Two sets of results (experimental validation part) are also provided as supplementary material.

## Abbreviations

*B*: Nucleation rate (#/m^3^ s) in Eq. 1
*C*: Concentration of solute (kg/m^3^)
*C**: Solubility or saturation concentration (kg/m^3^)
*ΔC*: Supersaturation (kg/m^3^) = C–C*
*D*, *D*_*m*_: Diffusion coefficient or laminar diffusivity (m^2^/s)
*D*_*t*_: Turbulent diffusivity (m^2^/s)
*f*: Number density function (#/m_c_ m^3^)
*f*_*r*_: Derivative of number density function (#/m_c_^2^ m^3^)
*f*_*w*_: Mass density function (kg/m_c_ m^3^)
*f*_*ϕ*_: Joint probability function of all scalars
g: Gravitational acceleration (m/s^2^)
*G*_*r*_: Growth rate (m/s)
*N*_*r*_: Nucleation rate (#/m^3^ s]
*G*_(__*p*__)_: Rate of change of *p* = (*p*1 *p*2 *… pNe*) due to micro-mixing
*G*_*s*__(__*p*__)_: Term to eliminate spurious dissipation rate
*h*: Enthalpy per unit mass (J/kg)
*H*: Numerical flux
*k*: Turbulent kinetic energy (m^2^/s^2^) in turbulence and micro-mixing equations
*k*_*v*_: Volume shape factor (−)
*M*^*n*^: Rate of change of $s_{n}$ due to micro-mixing
$M_{s}^{n}$: Term to eliminate spurious dissipation rate
*N*: Number of particle size cells or bins
*N*_*e*_: Number of probability modes or environments
*P*: Pressure (Pa) in momentum conservation equation
*P*_*n*_: Probability of mode *n* or volume fraction of Environment *n* in micro-mixing model
*r*: Crystal size (m)
*r*_0_: Nuclei size (m)
*Δr*: Discretized bin size for crystal size (m)
*Re*: Reynolds number
$s_{n}$: Weighted concentration of mean composition of scalars ***ϕ*** in mode *n*
*S*: Supersaturation ratio = c/c*
*S*_*as*_: User defined source term of anti-solvent concentration (kg/m^3^ s)
*t*: Time (s)
*T*: Temperature (k)
$$\vec{v}$$: Velocity vector (m/s)
*W*_*as*_: Anti-solvent mass percent (%)
*G*_*k*_: Generation kinetic energy of turbulence caused by velocity gradient
*ε*: Turbulent kinetic energy dissipation rate (m^2^/s^3^)
$\varepsilon_{\xi }$: Scalar dissipation rate (1/s)
$\xi^{\prime 2}$: Mixture fraction variance
$$\xi$$: Mixture fraction
$\Phi$: Mean composition of scalar in environment
$\rho_{3}$: Fluid density of Environment 3
$$\mu$$: Viscosity (kg/m s)
$\mu_{t}$: Turbulent viscosity (kg/m s)
$\theta$: Constant in minmod limiter
$$\rho$$: Density (kg/m^3^)
$\rho_{c}$: Crystal density (kg/m^3^)
$\tau$: Stress tensor (kg/m s^2^)
$$v$$: Kinematic viscosity (m^2^/s)

## Subscripts

*c*: Crystal property
*i*: Crystal dimension in population balance equation
*j*: Discretized bin for crystal size in population balance equation
*n*: Environment in micro-mixing model
