# Supplementary material for: Computational fluid dynamics simulation of a jet crystallizer for continuous crystallization of lovastatin
Source: Sci Rep. 2024 Jan 9;14:907. doi: 10.1038/s41598-023-51088-y (PMC10776609; doi:10.1038/s41598-023-51088-y)
Supplement: Supplementary file 3 — Supplementary Information 3. [file 41598_2023_51088_MOESM3_ESM.docx]

Guide for supplementary files:

**CaseHighVelocity_Exp.zip:** The simulation results (at t = 3.5 s) of jet crystallizer corresponding to experimental data at high jet velocity (refer to Fig. 3 in the manuscript).

**CaseLowVelocity_Exp.zip:** The simulation results (at t = 3.5 s) of jet crystallizer corresponding to experimental data at high jet velocity (refer to Fig. 3 in the manuscript).

The simulation results in Fig. 3 are obtained from these files. You can visualize the results using Paraview® software. It is free to download on windows and linux operating system.
